# Genomic epidemiology of the rotavirus G2P[4] strains in coastal Kenya pre- and post-rotavirus vaccine introduction, 2012–8

**DOI:** 10.1093/ve/vead025

**Published:** 2023-04-15

**Authors:** Timothy O Makori, Joel L Bargul, Arnold W Lambisia, Mike J Mwanga, Nickson Murunga, Zaydah R de Laurent, Clement S Lewa, Martin Mutunga, Paul Kellam, Matthew Cotten, D James Nokes, My Phan, Charles N Agoti

**Affiliations:** Epidemiology and Demography Department Kenya Medical Research Institute (KEMRI)-Wellcome Trust Research Programme, Off Hospital Road, P.O BOX 230-80108, Kilifi, Kenya; Department of Biochemistry, Jomo Kenyatta University of Agriculture and Technology, Kalimoni, PO Box 62000-00200, Juja, Kenya; Department of Biochemistry, Jomo Kenyatta University of Agriculture and Technology, Kalimoni, PO Box 62000-00200, Juja, Kenya; International Centre of Insect Physiology and Ecology, Animal Health Theme, ICIPE Road Kasarani, P.O BOX 30772-00100, Nairobi, Kenya; Epidemiology and Demography Department Kenya Medical Research Institute (KEMRI)-Wellcome Trust Research Programme, Off Hospital Road, P.O BOX 230-80108, Kilifi, Kenya; Epidemiology and Demography Department Kenya Medical Research Institute (KEMRI)-Wellcome Trust Research Programme, Off Hospital Road, P.O BOX 230-80108, Kilifi, Kenya; Epidemiology and Demography Department Kenya Medical Research Institute (KEMRI)-Wellcome Trust Research Programme, Off Hospital Road, P.O BOX 230-80108, Kilifi, Kenya; Epidemiology and Demography Department Kenya Medical Research Institute (KEMRI)-Wellcome Trust Research Programme, Off Hospital Road, P.O BOX 230-80108, Kilifi, Kenya; Epidemiology and Demography Department Kenya Medical Research Institute (KEMRI)-Wellcome Trust Research Programme, Off Hospital Road, P.O BOX 230-80108, Kilifi, Kenya; Epidemiology and Demography Department Kenya Medical Research Institute (KEMRI)-Wellcome Trust Research Programme, Off Hospital Road, P.O BOX 230-80108, Kilifi, Kenya; Department of Infectious Diseases, Faculty of Medicine, Imperial College London, Exhibition Road, London SW7 2AZ, UK; Kymab Ltd, The Bennet Building (B930), Babraham Research Campus, Cambridge CB22 3AT, UK; Medical Research Centre (MRC)/Uganda Virus Research Institute, Plot No: 51-59 Nakiwogo Road, P.O.Box 49, Entebbe, Uganda; MRC-University of Glasgow, Centre for Virus Research Glasgow, 464 Bearsden Road, Glasgow G61 1QH UK; Epidemiology and Demography Department Kenya Medical Research Institute (KEMRI)-Wellcome Trust Research Programme, Off Hospital Road, P.O BOX 230-80108, Kilifi, Kenya; School of Life Sciences and Zeeman Institute (SBIDER), The University of Warwick, Gibbet Hill Campus, Coventry CV4 7AL, UK; Medical Research Centre (MRC)/Uganda Virus Research Institute, Plot No: 51-59 Nakiwogo Road, P.O.Box 49, Entebbe, Uganda; MRC-University of Glasgow, Centre for Virus Research Glasgow, 464 Bearsden Road, Glasgow G61 1QH UK; Epidemiology and Demography Department Kenya Medical Research Institute (KEMRI)-Wellcome Trust Research Programme, Off Hospital Road, P.O BOX 230-80108, Kilifi, Kenya; School of Health and Human Sciences, Pwani University, Kilifi-Malindi Road, P.O BOX 195-80108, Kilifi, Kenya

**Keywords:** rotavirus group A, G2P[4], coastal Kenya

## Abstract

The introduction of rotavirus vaccines into the national immunization programme in many countries has led to a decline in childhood diarrhoea disease burden. Coincidentally, the incidence of some rotavirus group A (RVA) genotypes has increased, which may result from non-vaccine-type replacement. Here, we investigate the evolutionary genomics of rotavirus G2P[4] which has shown an increase in countries that introduced the monovalent Rotarix® vaccine. We examined sixty-three RVA G2P[4] strains sampled from children (aged below 13 years) admitted to Kilifi County Hospital, coastal Kenya, pre- (2012 to June 2014) and post-(July 2014 to 2018) rotavirus vaccine introduction. All the sixty-three genome sequences showed a typical DS-1-like genome constellation (G2-P[4]-I2-R2-C2-M2-A2-N2-T2-E2-H2). Pre-vaccine G2 sequences predominantly classified as sub-lineage IVa-3 and co-circulated with low numbers of sub-lineage IVa-1 strains, whereas post-vaccine G2 sequences mainly classified into sub-lineage IVa-3. In addition, in the pre-vaccine period, P[4] sub-lineage IVa strains co-circulated with low numbers of P[4] lineage II strains, but P[4] sub-lineage IVa strains predominated in the post-vaccine period. On the global phylogeny, the Kenyan pre- and post-vaccine G2P[4] strains clustered separately, suggesting that different virus populations circulated in the two periods. However, the strains from both periods exhibited conserved amino acid changes in the known antigenic epitopes, suggesting that replacement of the predominant G2P[4] cluster was unlikely a result of immune escape. Our findings demonstrate that the pre- and post-vaccine G2P[4] strains circulating in Kilifi, coastal Kenya, differed genetically but likely were antigenically similar. This information informs the discussion on the consequences of rotavirus vaccination on rotavirus diversity.

## Introduction

In 2009, the World Health Organization (WHO) recommended inclusion of rotavirus vaccines into the national immunization programmes (NIPs) globally (WHO 2021). Kenya introduced the WHO pre-qualified Rotarix® rotavirus group A (RVA) vaccine into its NIP in July 2014 (Varghese, Kang, and Steele 2022). With the increasing uptake of rotavirus vaccines globally, there has been a significant reduction in RVA-associated disease burden, but this virus still caused about 128,500 deaths in 2016 alone (Tate et al. 2016; Troeger et al. 2018b), with the majority of cases occurring in low-income countries (Troeger et al. 2018a). In Kenya, post-vaccine introduction impact studies have reported remarkable Rotarix® vaccine coverage of between 84 and 92 per cent by 2017 and a significant reduction of rotavirus-associated diarrhoea hospitalization of about 80 per cent (95 per cent confidence interval, 46–93) in the second-year post-vaccination, both in children under 5 years (Otieno et al. 2020), a vaccine effectiveness of ∼64 per cent (Khagayi et al. 2020), and a significant increase in the positivity rate of Rotarix® heterotypic genotypes such as G2P[4] (7.0 vs. 20.7 per cent, *P* < 0.001) and G3 (1.3–16.1 per cent, *P* < 0.001) (Gikonyo et al. 2020; Mwanga et al. 2020). Similar findings were observed in other countries that introduced the Rotarix® vaccine in their NIPs (Zeller et al. 2010; Than, Jeong, and Kim 2014; Al-Ayed et al. 2017; Vizzi et al. 2017; Degiuseppe and Stupka 2018; Khandoker et al. 2018; Roczo-Farkas et al. 2018; Simwaka et al. 2018; Thanh et al. 2018; Carvalho-Costa et al. 2019).

Whole-genome analysis of RVA can reveal the transmission and evolutionary history of circulating strains, including emerging mutations, and the origins and genetic diversity of the strains circulating in a particular region (Ghosh and Kobayashi 2011). The rotavirus G2P[4] genotype is known to possess a DS-1-like genomic constellation (G2-P[4]-I2-R2-C2-M2-A2-N2-T2-E2-H2) (Matthijnssens et al. 2008). G2P[4] strains are believed to have undergone genetic evolution in a stepwise pattern (Doan et al. 2015). This is from lineage I to lineage IVa in the *NSP5, NSP1, VP2, VP4,* and *VP7* genome segments and from lineage I to lineage V in the *VP1, VP3, VP6, NSP2, NSP3*, and *NSP4* genome segments, with some of the strains undergoing intra-genotype reassortments in the *VP7, VP3,* and *NSP4* genome segments after 2004 giving rise to emergent lineages of V in the *VP7* segment, lineages VI and VII in the *VP3* segments, and VI, VII, VIII, IX, and X lineages in the *NSP4* genome segment (Giammanco et al. 2014; Doan et al. 2015; Agbemabiese et al. 2016). The G2P[4] lineages circulating in Kenya are unknown.

The characterization of South African G2P[4] strains, through comparison of strains occurring during pre-and post-introduction of the Rotarix® vaccine, revealed sub-lineage shifts from G2 sub-lineage IVa-1 to G2 IVa-3, and P[4] sub-lineage IVa to P[4] IVb, and these shifts in genetic evolution were attributed to arise due to natural fluctuations and not as a result of vaccine pressure (Mwangi et al. 2022). Similarly, whole-genome analysis of G2P[4] strains circulating in Ghana (Agbemabiese et al. 2016), Australia (Donato et al. 2014, 2021), South Korea (Thanh et al. 2018), Bangladesh (Aida et al. 2016), and Brazil (Gómez et al. 2014) showed that the implementation of Rotarix® vaccination does not influence the genetic diversity of the circulating G2P[4] strains and that common amino acid replacements in the VP7 antigenic epitopes, including A87T, D96N, and S213D were reported, irrespective of the vaccination period.

The main goal of this study was to conduct whole-genome analysis of the G2P[4] genotypes circulating in Kilifi County, coastal Kenya, to determine whether the introduction of the Rotarix® vaccine in Kenya’s NIP impacts the genetic diversity of the circulating G2P[4] viral populations. Data from this study were compared with contemporaneous global RVA strains to establish the phylogenetic context and potential origin of Kenya’s pre- and post-vaccination rotavirus G2P[4] strains.

## Materials and methods

### Ethical approval

We collected samples from children to screen for and determine the genetic diversity of RVA viral populations. The study was conducted with strict adherence to the study protocols approved by the Scientific and Ethics Review Unit (REFERENCE Nos. 3049 and 2861) at the Kenya Medical Research Institute (KEMRI), Nairobi, Kenya. Parents and guardians of the eligible children were provided with sufficient information about the research study to allow each individual to make informed and independent decisions for their children to be enrolled in the study. Sample collection proceeded after obtaining written informed consent from the parents and guardians.

### Study participants

**Figure 1.**
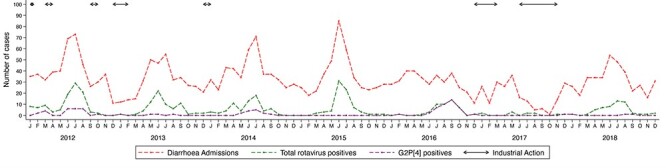
Temporal pattern of the cases of diarrhoea, rotavirus A, and G2P[4] genotype in KCH between 2012 and 2018. Determination of the G and P genotypes was done by partial segment sequencing using the Sanger approach.

The study was based at Kilifi County Hospital (KCH), a referral health facility that mainly serves the people of Kilifi County located in the North Coast of Kenya (Scott et al. 2012). The stool samples were obtained from children, aged below 13 years, who presented with diarrhoea as one of the illness symptoms and were admitted to KCH (Khagayi et al. 2020; Otieno et al. 2020). The study was undertaken in a paediatric ward at KCH, and the upper age of admission is 12 years. Although severe rotavirus-associated diarrhoea tends to occur in children aged under 5 years, rotavirus disease burden may continue well beyond this age group, and infections/reinfections are known to occur throughout a lifetime. There are reports of shifting the burden of severe rotavirus infection to older age groups because of the introduction of rotavirus vaccination into NIPs (Kyo et al. 2021). Thus, this study included a broad age range to be able to examine such recent claims if they have also occurred in the local population. Diarrhoea was defined as the passing of watery stool at least three times in the last 24 h. Overall, 2,690 children were enrolled during the study period (January 2012–December 2018) (Fig. 1). Of the 1,980 screened cases, 429 were positive for RVA, and 87 were confirmed as infected with G2P[4] strains (Fig. 1).

The stool samples were screened to detect RVA by using the enzyme-linked immunoassay kit (ProSPect™; Oxoid, Basingstoke, UK). All the RVA-positive samples were initially genotyped by a partial segment sequencing approach (Mwanga et al. 2020). *VP7* and *VP4* genes were sequenced, and G and P genotypes were inferred using the Virus Pathogen Resource (ViPR) tool for RVA (Pickett et al. 2012). Those classified as G2P[4] based on the outer capsid proteins were selected for this study (Fig. 1) (Mwanga et al. 2020).

### RNA extraction and processing

The samples collected in the pre-vaccine period (January 2012–June 2014) were sequenced using an agnostic whole-genome sequencing method (Phan et al. 2016), while the post-vaccine samples (July 2014–December 2018) were sequenced using an amplicon-based whole-genome sequencing approach (Fujii et al. 2012; Magagula et al. 2015).

Processing of the pre-vaccine samples was done by a centrifugation of 110 μl of stool suspension in phosphate-buffered saline for 10 min at 10,000 × g. Next, 2 U/μl of TURBO DNase (No. AM2238; Life Technologies, Carlsbad, CA, USA) was added to degrade non-encapsulated deoxyribonucleic acids (DNA). Nucleic acid extraction was performed according to the Boom method (Boom et al. 1990). First-strand cDNA was synthesized using the SuperScript III Reverse Transcriptase Kit (No. 18064014; Life Technologies, Carlsbad, CA, USA) with non-ribosomal random hexamer primers (Endoh et al. 2005). Second-strand cDNA was synthesized using 5 U of Klenow fragment 3ʹ–5ʹ exo- (No. M0212S; New England Biolabs, Ipswich, USA).

Processing of the post-vaccine introduction samples was done by subjecting 200 mg of stool specimens to bead beating (Liu et al. 2016), followed by nucleic acid extraction using the QIAamp Fast DNA Stool Mini Kit (No. 51604; Qiagen, Manchester, UK) following the manufacturer’s instructions. Reverse transcription–polymerase chain reaction (RT-PCR) was performed using the SuperScript IV One-Step RT-PCR System (No. 2594025, Thermo Fisher Scientific, Waltham, MA, USA) following the manufacturer’s instructions. The published primers used in our study to conduct RT-PCR assays were adopted from earlier studies (Supplementary Table S1) (Fujii et al. 2012; Magagula et al. 2015). The PCR conditions for the non-structural genome segments (*NSP1, NSP2, NSP3, NSP4,* and *NSP5*) consisted of forty cycles of thermocycling (30 s at 90°C, 1 min at 55°C, and 4 min at 68°C), whereas amplification of structural genome segments (*VP1, VP2, VP3, VP4, VP6,* and *VP7*) included forty cycles of thermocycling (90°C for 30 s, 61°C for 1 min, and 68°C for 6 min) and a final extension at 72°C for 4 min. PCR amplicons were resolved under a 2 per cent agarose gel stained with RedSafe (iNtRON Biotechnology, Inc.) for visualization of DNA bands. PCR products were purified using Exonuclease I (No. EN0581; Thermo Fisher Scientific, Waltham, MA, USA) as described by the manufacturer and pooled for each sample.

### Next-generation sequencing

The preparation of standard Illumina libraries for the pre-vaccine samples was performed according to the published protocol (Phan et al. 2016). Briefly, the double-stranded cDNA for each sample was sheared to obtain 400–500 nucleotide fragments. Each sample was then indexed separately to unique adapters and multiplexed at ninety-five samples and then sequenced on a HiSeq platform to generate about 1.5 million 250-bp paired-end reads per sample.

For the post-vaccine samples, pooled amplicons for each sample were purified using the Agencourt AMPure XP Kit (No. A63881; Beckman Coulter, USA) as described by the manufacturer. Library preparation was performed using the Illumina DNA flex (No. 20025519, Illumina, San Diego, CA, USA) as per the manufacturer’s specifications. Briefly, bead-linked transposomes were used to tagment the DNA, followed by the addition of adapters to the DNA fragments using a limited PCR programme. The adapter-linked DNA was cleaned using the tagment wash buffer. After that, the purified tagmented DNA was amplified via a limited-cycle PCR programme that adds the i7 and i5 adapters and sequences required for cluster generation during sequencing. Next, the amplified libraries were purified using a double-sided bead purification method. Subsequently, each DNA library was quantitated, and the correct insert sizes were confirmed on an Agilent 2100 Bioanalyzer using the Agilent high-sensitivity DNA kit (No. 5067; Agilent, Santa Clara, CA, USA). The DNA libraries were quantified on the Qubit Fluorimeter 2.0 using the Qubit dsDNA HS Assay Kit (No. Q32851, Life Technologies, Thermo Fisher Scientific, Waltham, MA, USA), normalized, and pooled at equimolar concentrations. Pooled DNA libraries were denatured and sequenced on the Illumina MiSeq platform (Illumina, San Diego, CA, USA) to generate 150 paired-end reads.

### Genome assembly

Quality trimming of Illumina FASTQ reads was done using Trimmomatic (Phred score: >30) with the following flags ‘ILLUMINACLIP: adapters_file: 2:30:10 LEADING:3 TRAILING:3 SLIDINGWINDOW: 4:15 MINLEN:36’ to remove adapters and low-quality bases (Bolger, Lohse, and Usadel 2014). *De novo* assembly of the quality trimmed reads was done using Spades with the following flags ‘-k 99,127 --careful’ (Bankevich et al. 2012). For the pre-vaccine sequences, RVA-specific contigs were identified using USEARCH (Edgar 2010) and a Sparse Linear Method algorithm (Cotten et al. 2014). Partial and overlapping contigs were joined using Sequencher (Gene Codes 2023) to obtain full-length sequences. For the post-vaccine sequences, Quast was used to check the quality of the contigs (Gurevich et al. 2013). Next, Artemis was employed to determine the open reading frames of each RVA segment (Carver et al. 2012). Then, genotyping of the assembled pre- and post-vaccine sequences was done using the ViPR tool for RVA (Pickett et al. 2012). The nucleotide sequences generated in this study have been deposited into GenBank under accession numbers MZ093788–MZ097268 and OP677569–OP677754 (Supplementary Table S2).

### Global sequences collection and processing

All available G2P[4] sequences irrespective of the sequence length and their corresponding metadata, including year of collection and location, were downloaded from the ViPR of RVA (Pickett et al. 2012). Records missing metadata were manually searched and any information, including location and collection year, that could be found in the primary publications was included in the respective sequence data. The sequences were subset to obtain datasets of each genome segment. The datasets of all the genome segments were filtered to only include samples with all the eleven segments. For all the eleven segments, more than 80 per cent of the coding sequence (CDS) region was considered for analysis. Overall, 350 global sequences for each segment met the inclusion criteria for phylogenetic analyses (Supplementary Table S2).

### Phylogenetic analysis

The global dataset was combined with the sequences of this study for each genome segment and aligned using MAFFT (v7.487) with the command ‘mafft --auto --reorder --preservecase input_file.fasta > output_file.fasta’ (Katoh et al. 2002). The maximum likelihood (ML) phylogenetic trees were reconstructed using IQTREE2 (v2.1.3) (Minh et al. 2020) using the best model selection (Subha et al. 2017) and 1,000 bootstrap replication settings (Hoang et al. 2018). The ML trees were linked to the respective metadata in R v4.1.0, and the ‘ggTree’ (https://github.com/YuLab-SMU/ggtree) R package was used to plot and visualize the trees (https://www.r-project.org/). For lineage designation, the sequences of previously described lineages for each segment (Giammanco et al. 2014; Doan et al. 2015; Agbemabiese et al. 2016) were utilized as the references (Supplementary Table S2).

### Selection pressure analysis

Analysis of selection pressure for the sixty-three Kilifi G2P[4] strains was performed using the tools in the DataMonkey webserver (fixed effects likelihood and Fast Unconstrained Bayesian AppRoximation (FUBAR); Murrell et al. 2013; Weaver et al. 2018). A site was considered to be under positive selection if it was detected by the two methods.

### Statistical analyses

All statistical analyses were carried out using Stata v13.1 (StataCorp 2021). The chi-squared test was used to compare among groups, with *P* < 0.05 indicating statistical significance.

## Results

### Baseline characteristics of the study participants

The peaks of RVA infections coincided with cases of diarrhoea during the study period between May and September (Fig. 1). However, the sample collection was majorly disrupted in 2016 and 2017 by strikes of health-care providers (Fig. 1) (Khagayi et al. 2020). One huge peak of G2P[4] infections was observed between June and November 2016 (post-vaccine), and a small one was documented between May and September 2012 (pre-vaccine) (Fig. 1).

No significant difference was reported in age, age groups, gender, and vaccination status between the cases infected with G2P[4] genotypes and the non-G2P[4] genotypes (*P* > 0.05) (Table 1). All the eighty-seven (20.3 per cent) G2P[4] samples genotyped by VP7 and VP4 Sanger sequencing (Mwanga et al. 2020) were taken forward to whole-genome sequencing; however, only sixty-three (14.7 per cent; thirty-two pre- and thirty-one post-vaccine periods) samples yielded full-genome sequences (Table 1). Of the sequenced samples, thirteen (20.6 per cent) were from children who received two doses of the Rotarix® vaccine (Table 1).

**Table 1.** Demographic characteristics of the study participants infected with RVA in Kilifi.

Characteristics	RVA positives (per cent)	Successfully sequenced G2P[4] (per cent)	All G2P[4] (per cent)	Non-G2P[4] (per cent)	All G2P[4] vs. non-G2P[4] *P*-value
Total cases	429	63 (14.7)	87 (20.3)	342 (79.7)	
Vaccine period					0.039
Pre-vaccine	215 (49.9)	32 (50.7)	35 (40.2)	180 (52.6)	
Post-vaccine	214 (50.1)	31 (49.3)	52 (59.8)	162 (47.4)	
Age (in months)					0.944
Mean (SD)	14.9 (13.1)	17.6 (16.4)	16.9 (15.4)	14.4 (12.4)	
Median (IQR)	11.7 (8.3–17.9)	11.2 (8.6–20.7)	10.6 (8.4–19.7)	12.2 (8.3–16.8)	
Age group (in months)					0.092
0–11	216 (50.4)	33 (52.4)	47 (54.0)	216 (50.4)	
12–23	169 (39.4)	20 (31.2)	26 (29.9)	143 (41.8)	
24–59	35 (8.2)	7 (11.1)	11 (12.6)	24 (7.0)	
≥60	9 (2.1)	3 (4.8)	3 (3.5)	6 (1.7)	
Gender					0.789
Male	251 (51.5)	37 (58.7)	52 (59.7)	199 (58.2)	
Vaccination status					0.129
Vaccinated	88 (20.6)	14 (22.2)	22 (25.6)	66 (19.4)	
Not vaccinated	215 (50.4)	33 (52.4)	35 (40.7)	180 (52.8)	
Unknown	124 (29.0)	16 (25.4)	29 (33.7)	95 (27.9)	
Full vaccination^a^	79 (18.4)	13 (20.6)	21 (24.1)	58 (17.0)	0.256

a Full vaccination implies that the participants received two doses of the Rotarix vaccine as recommended by the WHO.

### Genome constellations

To determine the genetic diversity in the Kilifi G2P[4] strains and their genetic relatedness with global strains, near full-genome sequences (>80 per cent genome coverage) of the sixty-three Kilifi samples were sequenced from the pre- (*n* = 32) and post- (*n* = 31) vaccine periods (Supplementary Table S2). Using the ViPR for the RVA genotype determination (Pickett et al. 2012), all the sixty-three sequences were classified as the G2-P[4]-I2-R2-C2-M1-A2-N2-T2-E2-H1 genotype (DS-1-like typical genome constellation) as shown in Supplementary Table S3.

### Phylogenetic and sequence analysis

**Figure 2.**
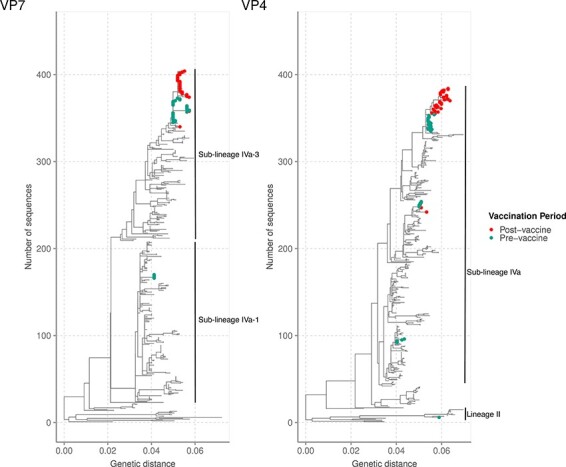
Phylogenetic reconstruction of the sixty-three Kilifi G2P[4] sequences against a backdrop of 350 global sequences for the VP7 and VP4 RVA genome segments using ML methods. The Kilifi sequences are coloured by the period of the sample collection (either before or after vaccine introduction in Kenya). For all the global sequences, more than 80 per cent of the CDS region was considered for analysis. For the Kilifi sequences, >80 per cent of the CDS region was used for the VP7 and 68 per cent for the VP4 segment. The study sequences were classified into the lineages indicated in each phylogenetic tree.

To gain insights into the genetic diversity of the study G2P[4] strains in the global context, genetic distance-resolved phylogenetic trees were constructed for all the eleven gene segments (Fig. 2; Supplementary Fig. S1). Sequence identity matrices of the study G2P[4] strains exhibited high nucleotide sequence similarities (93–100 per cent) in the *NSP1, NSP2, NSP3, NSP5, VP1, VP2, VP4, VP6,* and *VP7* genome segments and low to high nucleotide sequence similarity (85–100 per cent) in the *VP3* and *NSP4* genes (Table 2).

**Table 2.** The percentages of nucleotide similarity and amino acid identity for the Kilifi G2P[4] strains.^a^

Genome segment	Percentage of nucleotide similarity	Percentage of amino acid similarity
*VP4*	94.3–100	96.8–100
*VP7*	94.3–100	96.3–100
*VP6*	94.0–100	96.0–100
*VP1*	93.9–100	98.0–100
*VP2*	97.2–100	99.2–100
*VP3*	87.1–100	92.1–100
*NSP1*	95.7–100	96.0–100
*NSP2*	97.3–100	97.5–100
*NSP3*	97.1–100	98.4–100
*NSP4*	85.0–100	91.5–100
*NSP5*	94.5–100	94.5–100

a This table was generated by performing pairwise sequence analysis using ClustalW in MEGA v11.

### Analysis of the *VP7* gene

The *VP7* gene is highly variable and encodes the humoral immune response glycoprotein (Liu 2014). The *VP7* genetic distance-resolved phylogenetic tree showed that the Kilifi sequences formed three clusters: a monophyletic cluster, a minor monophyletic cluster, and a singleton (Fig. 2). Within the major cluster, the Kilifi strains separated by the vaccination period, with one sub-cluster consisting of strains circulating 2 years after Rotarix® vaccine introduction and were interspersed with three strains isolated from children admitted to Kenyatta National Hospital (KNH), Kenya, in 2017 (Fig. 2). These sequences shared two non-synonymous amino acid substitutions (S72G and S75L) with respect to the pre-vaccine strains (Supplementary Table S4). The second sub-cluster mainly consisted of strains circulating in the pre-vaccine period and two strains that circulated in July 2014, i.e. the early post-vaccine period (Fig. 2). The sequences in the minor monophyletic cluster consisted of five Kilifi strains collected in 2012, while the singleton Kilifi strain (KLF1033/2018) clustered with three strains detected in Mozambique in 2013 (Fig. 2).

**Figure 3.**
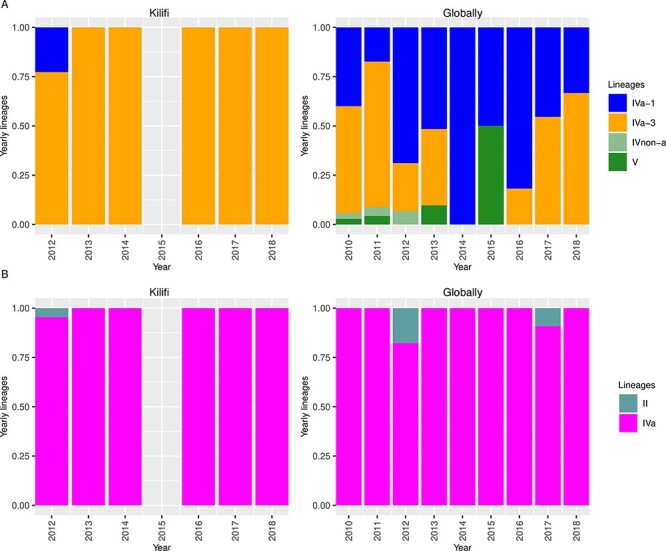
Temporal pattern of the G2 and P[4] lineages observed in Kilifi and globally. (A) Temporal pattern of the Kilifi G2 lineages from 2012 to 2018 and temporal pattern of the global G2 lineages from 2010 to 2018. (B) Temporal pattern of the Kilifi P[4] lineages from 2012 to 2018 and temporal pattern of the global P[4] lineages from 2010 to 2018.

With regard to the *VP7* lineages, the Kilifi G2 strains were classified into lineage IV and further classified into sub-lineages IVa-1 and IVa-3 (Fig. 2). Sub-lineages IVa-1 and IVa-3 sequences co-circulated in Kilifi in 2012, while sub-lineages IVa-1, IVa-3 and IV non-a were in circulation in the global context(Fig. 3A). However, IVa-1 strains in Kilifi were replaced with sub-lineage IVa-3 strains in 2013 that dominated until 2018 (Fig. 3A), unlike in the global context where sub-lineages IVa-1, IVa-3, and V co-circulated in 2013, sub-lineage IVa-1 predominantly circulated in 2014, sub-lineages IVa-1 and V co-circulated in 2015, and sub-lineage IVa-3 re-emerged in 2016 replacing lineage V and co-circulated with sub-lineage IVa-1 until 2018 (Fig. 3A). No lineage shift was observed pre- and post-vaccine introduction.

### Analysis of the *VP4* gene

The *VP4* gene is highly variable and encodes a highly immunogenic protease-sensitive protein involved in receptor binding and cell penetration (Liu 2014). In the *VP4* phylogenetic tree, the P[4] Kilifi sequences formed clusters (*n* > 2) mainly based on the vaccination period separated from global sequences (Fig. 2). However, two Kilifi sequences formed singletons, with the KLF1033/2018 strain clustering with a sequence isolated from a child admitted to KNH, while the KLF0616/2012 strain was interspersed with sequences from Mozambique (Fig. 2). A major cluster of Kilifi sequences further sub-clustered based on the vaccination period, with the post-vaccine sequences interspersing with Kenyan sequences isolated from children admitted to KNH (Fig. 2). In addition, Kilifi strains collected in 2014 and some 2012 strains formed two distinct clades, clustering separately from global sequences (Fig. 2).

During the study period, G2 lineage II and IVa strains circulated in Kilifi, with lineages II and IV co-circulating in 2012, consistent with the global context (Fig. 3B). From 2013 to 2018, sub-lineage IVa predominantly circulated in Kilifi similar to the global context (Fig. 3B). However, in the global context, few lineage II strains co-circulated with IVa strains in 2017 (Fig. 3B). No lineage shift was observed pre- and post-vaccine introduction (Fig. 2).

### Analysis of the backbone genome segments

The backbone genome segments of the Kilifi G2P[4] strains (*VP6, VP1-VP3*, and *NSP1–NSP5*) formed up to four clusters on the global phylogenetic trees (Supplementary Fig. S1). In the *VP6, VP1, VP2, VP3, NSP1,* and *NSP2* genes, the majority of the Kilifi sequences formed one major cluster which further separated into two sub-clusters of only pre- and post-vaccine sequences (Supplementary Fig. S1). The post-vaccine strains in these genes clustered closely with 2017 sequences from KNH, Kenya (Supplementary Fig. S1). In addition, the Kilifi 2014 sequences in the *VP6, VP3, NSP1,* and *NSP2* segments exhibited a different clustering pattern of a further minor sub-cluster irrespective of the vaccination period, consistent with the *VP4* gene (Supplementary Fig. S1). Four post-vaccine sequences (KLF1068, KLF0831, KLF0836, and KLF1078) were interspersed with the pre-vaccine sequences in the *NSP4* phylogeny, and a single post-vaccine sequence (KLF1078) clustered with pre-vaccine sequences in the *NSP3* phylogeny exhibiting high nucleotide sequence similarity (100 per cent) (Supplementary Fig. S1). The *NSP5* post-vaccine sequences formed one cluster, while the pre-vaccine sequences exhibited a different clustering pattern consisting of three distinct clusters (*n* ≥ 2) and three singletons (KLF0601/2012 was interspersed with a sequence from KNH, KLF1066/2014 and KLF0722/2014) (Supplementary Fig. S1). A minor cluster consisting of five Kilifi pre-vaccine strains (KLF0550/2012, KLF0551/2012, KLF0553/2012, KLF0558/2012, and KLF1064/2012) separate from global sequences was observed in the *VP1, VP2, VP3, NSP2, NSP4*, and *NSP5* genes; however, these strains were interspersed with sequences that circulated between 2012 and 2017 in Japan, Hungary, Australia, and Belgium in the *NSP3* gene and further formed two sub-clades in the *VP6* gene (Supplementary Fig. S1). In addition, a minor cluster of the Kilifi pre-vaccine sequences (KLF0640/2013, KLF0673/2013, and KLF0657/2013) interspersed with sequences from Malawi was observed in the *VP3* gene, indicating possible importation of these strains (Supplementary Fig. S1). Besides, a singleton of a post-vaccine sequence (KLF1033/2018) was interspersed with sequences from Mozambique that circulated in 2013 across all the backbone genes (Supplementary Fig. S1). For the *NSP5* gene, three other singletons were observed: KLF0601/2012 interspersed with a sequence from KNH, KLF1066/2014, and KLF0722/2014 each separate from global sequences (Supplementary Fig. S1). No lineage shifts were reported in the backbone genome segments (Supplementary Fig. S1).

The Kilifi G2P[4] strains shared low nucleotide (75–87 per cent) and amino acid (53–92 per cent) similarities with the Rotarix vaccine-derived strains (Table 3).

**Table 3.** The percentages of nucleotide similarity and amino acid identity for the Kilifi G2P[4] strains relative to the Rotarix-derived strains.^a^

Protein	Percentage of nucleotide similarity	Percentage of amino acid similarity
VP7	73	72–74
VP4	84–87	87–89
VP6	78–80	91–92
VP1	79	89–90
VP2	81–82	89–91
VP3	75–76	53–55
NSP1	75	68–69
NSP2	80–82	89–90
NSP3	77–78	82–83
NSP4	78–80	80–84
NSP5	84–85	80–83

a This table was generated by performing the pairwise sequence analysis using ClustalW in MEGA v11.

### Amino acid changes in the VP7 glycoprotein (G) and VP4 protease-sensitive (P) proteins

**Figure 4.**
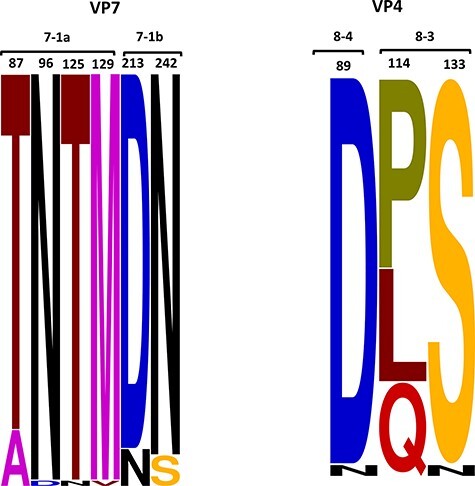
A sequence logo showing the amino acid changes observed in known antigenic epitopes of G2 and P[4] proteins in the Kilifi G2P[4] relative to the ancestral DS-1 strain. The changes in amino acids are shown in different colour schemes.

The pattern of amino acid substitutions in the G and P proteins was analysed in relation to the ancestral DS-1 sequence, the Rotarix vaccine strain, and vaccine-derived strains. The VP7 gene contains 7-1 (7-1a and 7-1b) and 7-2 antigenic epitopes, which affect the ability of antibodies to neutralize virus infectivity and reduce vaccine effectiveness (Aoki et al. 2009). The Kilifi strains exhibited seventeen amino acid changes relative to the DS-1 VP7 ancestral sequence (Supplementary Table S4). All the Kilifi strains had three amino acid (aa) mutations (D96N, N125T, and V129M) in the 7-1a antigenic epitope with respect to the DS-1 ancestral strain (Fig. 4; Supplementary Table S4). Furthermore, except for the 2014 sequences, the Kilifi sequences exhibited an A87T aa mutation in the 7-1a epitope (Supplementary Table S4). Compared to the DS-1 sequence, the Kilifi sub-lineage IVa-1 strains exhibited the N242S aa mutation, whereas sub-lineage IVa-3 strains harboured the N213D aa mutation: both in the 7-1b epitope (Supplementary Table S4). Besides, the I44M aa change was observed in the T lymphocyte epitope (40-52) of all the Kilifi strains (Supplementary Table S4). Compared with the Rotarix vaccine-derived strains, the majority of Kilifi pre- and post-vaccine period sequences showed matches at epitopes 7-1a (Position T87) and 7-1b (Position N213) (Supplementary Table S4).

The VP4 surface protein is cleaved into the VP8* and the VP5* domains containing the 8-1 to 8-4 and 5-1 to 5-5 antigenic epitopes (Dormitzer et al. 2002). Analysis of the VP4 aa mutations revealed that the Kilifi strains differed only at three positions: Q114P or L114P and N133S in the 8-3 epitope and N89D in the 8-4 epitope, relative to the DS-1 prototype sequence (Fig. 4; Supplementary Table S5). Three Kilifi pre-vaccine and twenty-seven post-vaccine period strains had one amino acid match at epitope 8-3 (Position P114) relative to the Rotarix vaccine-derived strains (Supplementary Table S5). There were no amino acid differences between sequences from vaccinated children and those from non-vaccinated children in both VP7 and VP4 antigenic epitopes.

### Selection pressure in the eleven genes

All the eleven genes were under purifying pressure (Table 4). No site was undergoing positive pressure selection (Table 4).

**Table 4.** Amino acid changes in the Kilifi post-vaccine strains relative to the pre-vaccine strains and the analysis of selection pressure.

			Selection pressure analysis	
Gene	Non-synonymous amino acid changes	Synonymous amino acid changes	Positive selection	Negative selection
*VP7*	S72G and S75L	None	0	12
*VP4*	S7R	F417, A451, and F527	0	26
*VP6*	G13D	None	0	18
*VP1*	Q959R	A157, S389, and R932	0	57
*VP2*	S233N, S416L, L658P, and Y792C	R831	0	52
*VP3*	N301D and P347S	T110, S132, Y622, V764, and S819	0	104
*NSP1*	D403N	I90	0	47
*NSP2*	K92R	None	0	9
*NSP3*	None	N16 and L223	0	21
*NSP4*	K59R	P34	0	19
*NSP5*	N141S	None	0	5

## Discussion

We investigated the evolutionary dynamics of G2P[4] strains sampled from children admitted to KCH, coastal Kenya, between 2012 and 2018. All the recovered genomes showed a typical DS-1-like constellation consistent with findings from several countries using the Rotarix® vaccine in their NIPs (Dennis et al. 2014; Doan et al. 2015; Agbemabiese et al. 2016; Mwangi et al. 2022).

Separate clusters of pre- and post-vaccine sequences were observed in the *VP1*-*VP4*, *VP6, VP7*, *NSP1, NSP2*, and *NSP5* segments. However, the strains sampled in July 2014 (the early post-vaccine period) clustered with pre-vaccine strains across all the eleven gene segments in our study possibly because the vaccine coverage was low and thus had no impact yet on the circulating genotypes and lineages. Unique clusters of either G2P[4] or G1[P8] strains separated by the RVA vaccine period have been reported in South Africa (Mwangi et al. 2022), Rwanda (Rasebotsa et al. 2020), Australia, and Belgium (Zeller et al. 2015), which were interpreting as reflecting natural genetic fluctuations rather than vaccine-induced evolution. However, the study Kilifi *NSP4* and *NSP3* genes exhibited some clusters of mixed pre- and post-vaccine sequences, indicating that in some strains some pre-vaccine genes persisted in circulation into the post-vaccine period.

Phylogenetic analyses indicated that the diversity of the Kilifi G2P[4] strains may have been locally restricted both in the pre- and post-vaccine periods, since Kilifi sequences clustered away from global sequences in all the eleven genome segments. In addition, limited sequence data from Kenya and East Africa may have contributed to this uncertainty about the regional context of Kilifi diversity. The Kilifi post-vaccine strains only clustered with Kenyan sequences sampled from children admitted to KNH in 2017, further suggesting locally restricted genetic evolution. However, one sample (KLF1033/2018) consistently clustered with sequences from Mozambique, and some pre-vaccine strains were interspersed with global strains for the *NSP3* segment, suggestive of limited introduction from other countries. No lineage shift was observed during the pre- and post-vaccine periods in Kilifi inconsistent with findings in South Africa (Mwangi et al. 2022), where RVA vaccine introduction was associated with lineage shift. Furthermore, few G2 and P[4] lineages were in circulation within Kilifi compared with the combined global data during the study period. This supported our hypothesis that local drivers were responsible for the diversity within the Kilifi setting. Despite the locally restricted diversity of the Kilifi G2P[4] strains, the observed lineages (G2 IVa-1 and IVa-3 and P[4] IV and II) have been reported in many countries globally (e.g. South Africa, Ghana, Australia, the USA) irrespective of the vaccination period (Dennis et al. 2014; Doan et al. 2015; Agbemabiese et al. 2016; Mwangi et al. 2022). In addition, only G2 lineage IVa-3 and P[4] lineage IVa were in circulation post-vaccine introduction suggesting reduced diversity. This is consistent with the findings of a global study (Hoxie and Dennehy 2021) where vaccine introduction coincided with a sharp decline in the genetic diversity of globally circulating RVA strains despite the different timescales of the two studies.

The Kilifi strains harboured six conserved amino acid substitutions in the VP7 antigenic epitopes: 7-1a and 7-1b with respect to the ancestral DS-1 G2P[4] strain. Three of these positions (A87T, D96N, and N213D) are critical for antibody binding, and sequence changes here may lead to escape from host neutralizing antibodies (Dyall-Smith et al. 1986). The I44M aa change may affect cellular immunity as this region harbours a known T lymphocyte epitope (40-52) of the VP7 genes. All Kilifi strains had this change that potentially resulted in the loss of recognition by T cells leading to escape from host immune responses (Wei et al. 2009; Morozova et al. 2015). Three amino acid changes were observed in VP4 antigenic epitopes in 8-4 (N89D) and 8-3 (Q114P or L114P and N133S) in the Kilifi strains. These have been associated with escape of attachment of the virus to host neutralizing monoclonal antibodies (Monnier et al. 2006). These amino acid substitutions were present in both pre- and post-vaccine Kilifi strains, suggesting that they were not brought about by vaccine use. The occurrence of few amino acid matches in the VP7 (T87 and N213) and VP4 (P114) antigenic epitopes between the Rotarix vaccine-derived strains and both Kilifi pre- and post-vaccine strains shows that vaccine pressure did not induce significant antigenic changes. The absence of amino acid differences in the VP7 and VP4 antigenic epitopes in post-vaccine sequences from vaccinated and non-vaccinated children suggested that the breakthrough infections in vaccinated children were from the prevalent clade that was also infecting the non-vaccinated children.

The consensus selection pressure in the coding regions across the eleven genes of the study strains was purifying pressure, which may be a strategy to remove any deleterious mutations arising from the error-prone RNA-dependent RNA polymerase (Liu 2014). Lack of positive selection in the codon sites further supports that the Kilifi strains were not under vaccine-induced pressure similar to findings in G2P[4] strains circulating in the south post-vaccine introduction (Mwangi et al. 2022).

This study had limitations. First, only sequences sampled from hospitalized children were analysed and thus may not conclusively reflect the diversity that was in circulation in the entire coastal Kenya population. We only analysed a few genomes across the years. Second, we only recovered near-complete genomes. Only 68 per cent coverage was recovered in the VP4 segment. Lastly, the global context genomes were a collection across many countries (i.e. Australia, Ghana, the USA, Japan, and Italy), some of which may have introduced rotavirus vaccination into their NIP, others not. The genomes used as part of the global context were obtained from a public sequence database (GenBank) and lacked information on the vaccination status of the host; therefore, we could not compare our sequences from vaccinated children with global sequences from vaccinated children.

In conclusion, our study reinforces the significance of genomic sequencing in monitoring the effect of vaccine pressure on circulating RVA strains in Kenya. The Kilifi strains to a large extent clustered based on the vaccination period and were separate from the global strains. Furthermore, conserved aa mutations were observed in the VP7 and VP4 antigenic epitopes of the pre- and post-vaccine strains, suggesting that the Rotarix® vaccine did not have a direct impact on the evolution of the circulating strains.

## Supplementary Material

vead025_Supp

## Data Availability

The epidemiological data are available on the Virus Epidemiology and Control (VEC) dataverse (https://doi.org/10.7910/DVN/P4MRVF).
