# Supplementary figures and images for: Genomic epidemiology of the rotavirus G2P[4] strains in coastal Kenya pre- and post-rotavirus vaccine introduction, 2012–8
Source: Virus Evol. 2023 Apr 15;9(1):vead025. doi: 10.1093/ve/vead025 (PMC10190042; doi:10.1093/ve/vead025)

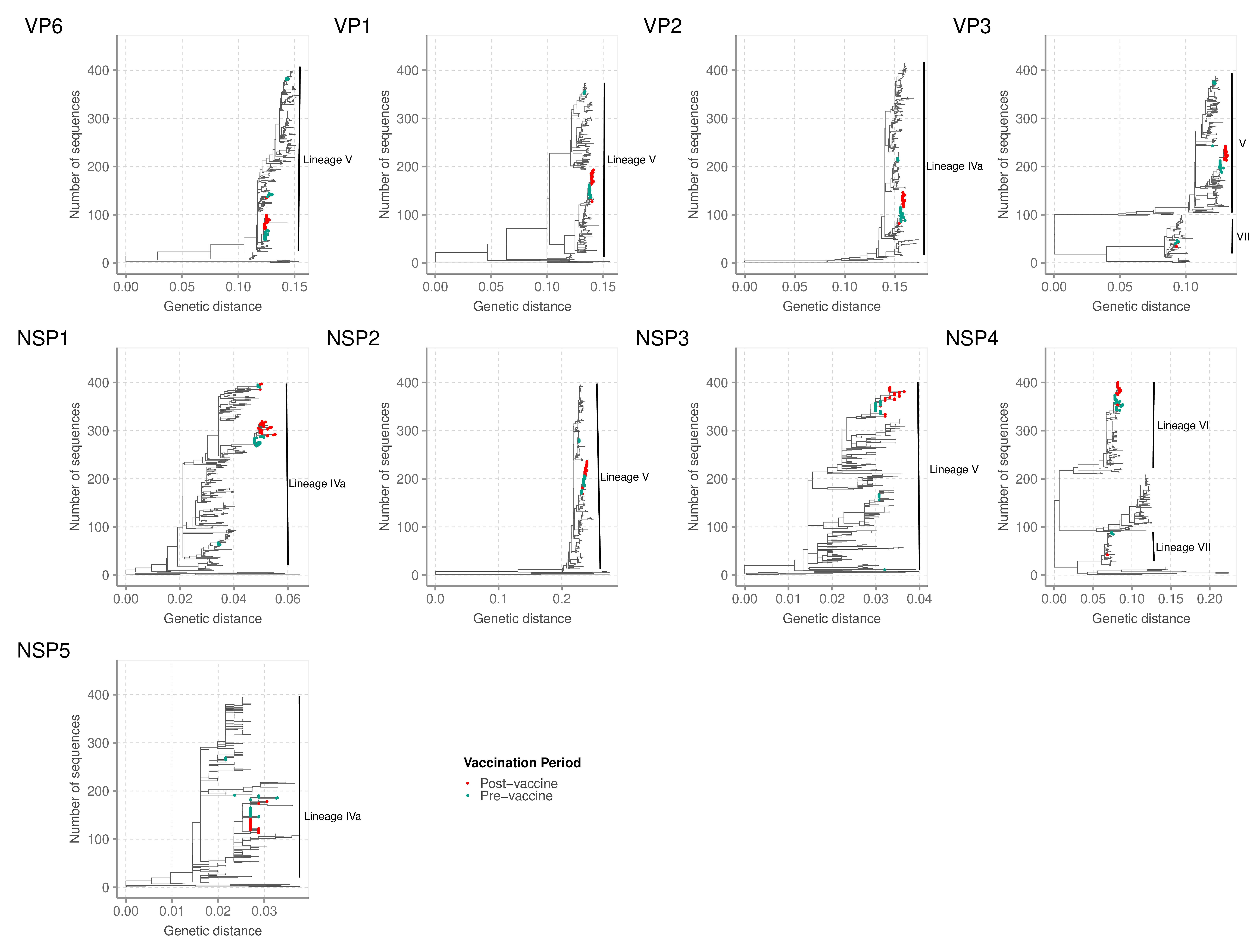

Supplement: vead025_Supp [file vead025_supp.zip › Fig S1.tiff]

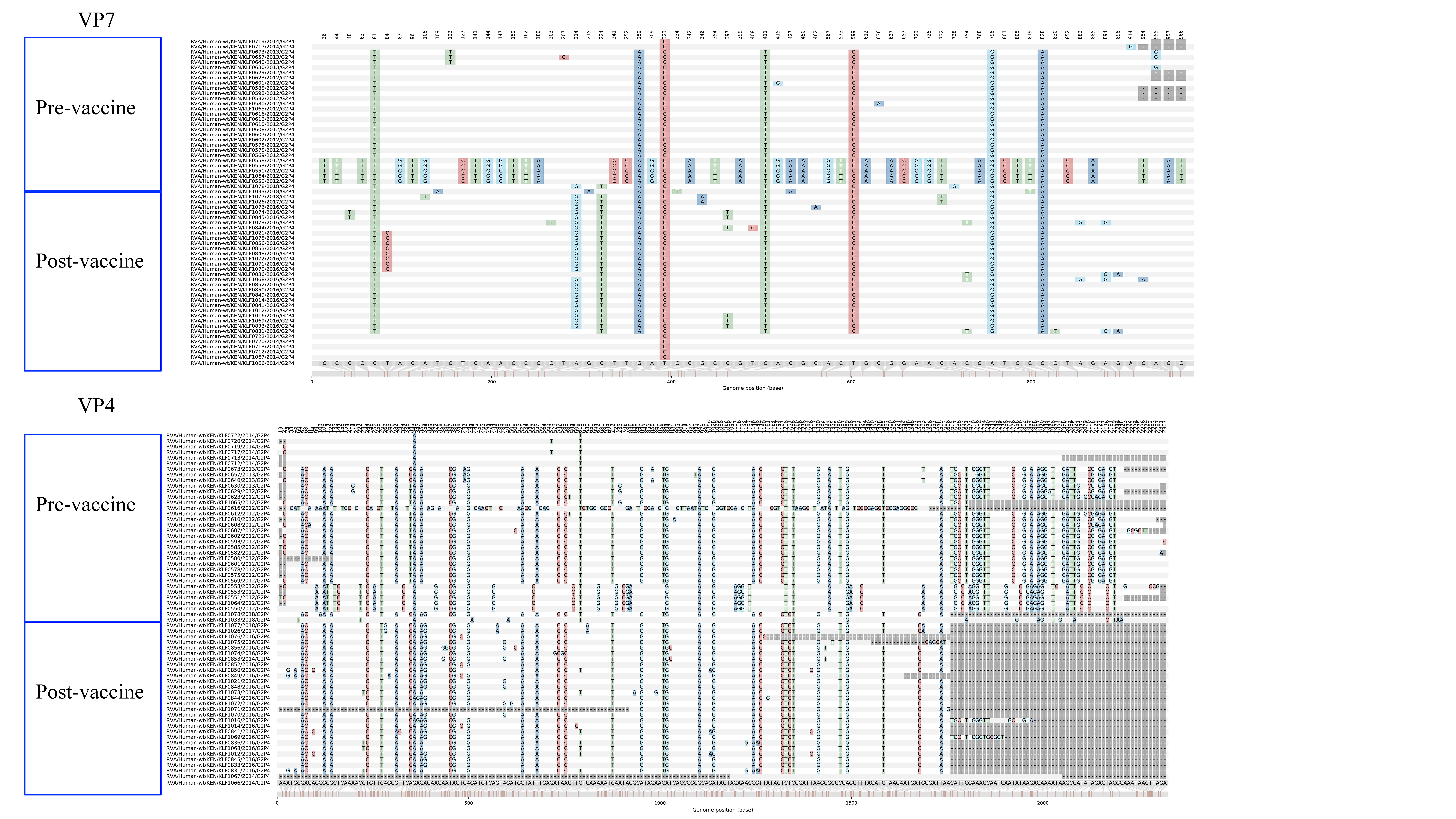

Supplement: vead025_Supp [file vead025_supp.zip › Fig S2.tiff]
